# Unmet basic needs negatively affect health-related quality of life in people aging with HIV: results from the Positive Spaces, Healthy Places study

**DOI:** 10.1186/s12889-018-5391-z

**Published:** 2018-05-21

**Authors:** Phan Sok, Sandra Gardner, Tsegaye Bekele, Jason Globerman, Mary V. Seeman, Saara Greene, Michael Sobota, Jay J. Koornstra, LaVerne Monette, Keith Hambly, Stephen W. Hwang, James Watson, Glen Walker, Sean B. Rourke, The Positive Spaces, Healthy Places Team

**Affiliations:** 1 Institute of Medical-Science, University of Toronto, Toronto, Canada; 2 Baycrest Health Sciences, Toronto, Canada; 3 Ontario HIV Treatment Network, Toronto, Canada; 4 Ministry of Health and Long-Term Care, Toronto, Canada; 5 School of Social Work, McMaster University, Hamilton, Canada; 6 AIDS Thunder Bay, Thunder Bay, Canada; 7 Bruce House, Ottawa, Canada; 8 Ontario Aboriginal HIV/AIDS Strategy, Toronto, Canada; 9 Fife House, Toronto, Canada; 10 Centre for Urban Health Solutions, St. Michael’s Hospital, Toronto, Canada; 11 Positive Living Niagara, St. Catherine, Canada; 12 Department of Psychiatry, University of Toronto, Toronto, Canada

**Keywords:** HIV, Aging, Basic needs, Health-related quality of life

## Abstract

**Background:**

Basic needs (e.g., food security and stable housing) are important determinants of health and well-being, yet their impact on health-related quality of life (HRQoL) in the context of HIV and aging has not been systematically investigated.

**Methods:**

Multiple linear regression models examined the relationship between unmet basic needs, and physical and mental HRQoL by age strata (20-34, 35-49 and 50+) in a cross-sectional sample of 496 people living with HIV in Ontario, Canada.

**Results:**

An overwhelming majority of participants (87%) reported unmet needs related to food, clothing or housing. The prevalence of unmet basic needs in the two older groups appeared to be lower than among younger participants, but the difference did not reach statistical significance. The presence of unmet basic needs predicted substantially lower mean physical health and mental health summary scores in the two oldest groups. Notably, age moderated the influence of unmet basic needs on HRQoL.

**Conclusions:**

The availability and accessibility of food security, appropriate clothing and stable housing for people living with HIV who are aging need to become a higher priority for program planners and decision makers.

## Background

Basic needs (e.g., food security and stable/affordable housing) are important determinants of health and well-being among people living with HIV. A study by Cunningham et al. [1] showed that when basic needs (food, clothing and housing) were unmet, more than one-third of HIV-positive people in the United States did not keep their medical appointments. Two other studies reported that unmet basic needs with respect to food, clothing and housing constituted the most important predictors of poor physical health and mental health in HIV-infected homeless women [2] and men [3]. A recent study showed that men living with HIV receiving community mental health supports had greater unmet health needs than HIV-negative men who did receive such supports [4].

Literature reviews of adults with HIV indicate that they face many challenges associated with health and well-being. These challenges encompass socioeconomics [5, 6], depression [7], housing [8] and food security [9, 10]. Older adults with HIV experience similar challenges [11–14]. In addition, they face issues of sexuality [15, 16], high risk behaviours [17, 18], premature age-related comorbidities [19], early frailty [20], poor physical function [21] and shortened life expectancy [22]. These added burdens contribute to negative health-related quality of life (HRQoL). We also know that the natural age process negatively impacts on physical health in the general population over age 65 [23] and on several dimensional subscales of HRQoL in people living with HIV [24]. What is still not clear is the degree to which unmet basic needs may impact HRQoL in the context of HIV and aging. This in critical as the effectiveness of combination antiretroviral therapy now allows people living with HIV to reach older age.

The objective of this study was to determine the degree to which unmet basic needs are associated with physical and mental HRQoL; specifically, whether these associations vary with increasing age. We hypothesized that unmet basic needs related to food, clothing or housing stability/affordability would significantly and negatively impact physical health and mental health quality of life among people living with HIV, especially with increasing age.

## Methods

### Study design and data

This cross-sectional study used baseline data (2006) from the Positive Spaces, Healthy Places (PSHP) study. The PSHP study is a five-year observational cohort of 602 individuals living with HIV in Ontario, Canada. It was designed to evaluate the effects of housing on health. We included 496 participants in the current study because 106 (17.6%) study participants were excluded due to missing data on one or more socioeconomic or clinical data points. Thirty-seven had no data on basic needs. We note that the overall mean physical health and mental health summary scores did not differ significantly between excluded and included participants. The PSHP study is supported by grants from the Canadian Institutes of Health Research (CBR-75568 and CBR-94036), the Ontario Ministry of Health and Long-Term Care, the Ontario AIDS Network, the Wellesley Institute and the Ontario HIV Treatment Network (CCB115).

### Procedures

To be eligible in the PSHP study, participants had to be HIV positive, 18-years or older, able to provide informed consent and reside in Ontario. To ensure people with unstable housing situations were included, participants were recruited from community-based AIDS service agencies, shelters, agencies serving women, Indigenous organizations, supportive housing agencies, transitional housing providers, agencies providing HIV programs and services for harder-to-reach populations (e.g., injection drug users, homeless people and those with unstable housing). All survey data were collected via face-to-face interviews conducted by trained peer researchers living with HIV. A 40 CAD honorarium was provided for participating. The study was approved by the Research Ethics Board at the University of Toronto (REB#25710). Additional details and descriptions of the PSHP cohort are available elsewhere [8].

### Measures

#### Conceptualizing basic needs

We conceptualized basic needs as those essential to daily life. We evaluated basic needs in three steps. Step 1, we looked at four items on the 62-item PSHP questionnaire relating to food, clothing, housing-related costs and risk of homelessness. For food, the participants were asked *“In the last 12 months, have you ever experienced difficulty in buying enough food?”* The response was *“yes* or *no”* (Question 60a). For clothing, the participants were asked: *“In the last 12 months, have you ever experienced difficulty in buying sufficient clothing for yourself or your dependent(s)?”* The response was *“yes* or *no”* (Question 61a). For housing-related costs, the participants were asked: *“Considering your income, how difficult is it for you to meet your monthly housing-related costs?”* The responses were *“very difficult, fairly difficult, very little difficult* or *not at all difficult”*. We, however, collapsed *“very difficult* and *fairly difficult”* into *“difficulty”* and collapsed *“very little difficulty* and *not at all difficult”* into *“non-difficulty”* (Question 23). For risk of homelessness, the participants were asked: *“How much do you pay in rent/mortgage every month?”* (Question 57). We adapted the Canadian Definition of Homelessness [25] that defined the risk of homelessness as occurring when a person is spending 30% or more of his or her income (before taxes) on rent or mortgage. A formula was created and applied to each participant in the sample^1^: $RH=\frac{inc}{mor\ }$. In step 2, we differentiated between *met* (coding = 0) and *unmet* (coding = 1). Thus, unmet basic needs were defined as responding either *yes* to food, or *yes* to clothing, or *difficulty* to housing-related costs or *being at risk* of homelessness. Finally, step 3, we tested for the internal consistency of the four items [26] Cronbach’s alpha was somewhat low, 0.58, but, because we tested only four items and the value of Cronbach’s alpha is affected by the number of items tested [27]. We felt that this was adequate for our purpose.

#### Outcome variables

We derived physical health and mental health summary scores from the 35-item Medical Outcomes Study HIV Health Survey (MOS-HIV) [28]. The MOS-HIV has been criticized [29] but is, nevertheless, considered to be a robust tool for measuring HRQoL in people living with HIV and has been translated into 19 languages [30]. The instrument includes 10 dimensions of health: general health (5 items), physical functioning (6 items), role functioning (2 items), social functioning (2 items), energy (4 items), mental health (5 items), health distress (4 items), cognitive functioning (4 items), pain (2 items) and quality of life (1 item). All scales were linearly transformed into a 0 (worst health) to 100 (best health) scale and were then converted into z-scores in order to standardize them to the reference population of patients with HIV/AIDS. We then computed and created standardized scores for physical health and mental health summary scores as per instructions of the survey developers [31]. In this study, the 35-item MOS-HIV had a Cronbach’s alpha of 0.90. Prior studies have suggested that HRQoL summary scores are reproducible, reliable and valid tools for measuring HIV patient functioning and well-being [32].

#### Age groups

We categorized participants into three age groups: young (20-34 years), middle- aged (35-49 years) and old (50+ years). We used the cut-off of age 50 for the beginning of *old age* as this cut-off is considered a clinically important threshold in this population [33].

#### Variables

Demographic variables included gender (female vs. male), ethnicity (Caucasian vs. other) and sexual orientation (heterosexual vs. other). Socioeconomic status variables included educational level (high school degree or above vs. no high school degree), employment status (employed vs. unemployed; unemployed included retired/disabled) and personal gross income (high vs. low income; low income meant below the cut-off of 1150 CAD/month**,** the median income of this study sample). Social factor variables included living arrangements (living with someone vs. living alone) and social support (high vs. low support; low support meant below the cut-off of 43, the median scores of this study sample). Social support scores were derived from the 19-item Medical Outcomes Study Social Support Survey [34]. HIV clinical marker variables included previous diagnosis of AIDS (absence vs. presence), CD4 T-cell count (above vs. below 200 cells/mL), years since HIV diagnosis and years since starting antiretroviral therapy. All variables were self-reported.

### Statistical analysis

#### Descriptive analyses

A descriptive analysis of sample characteristics was performed across three age strata. For categorical comparisons, a Chi-square (χ^2^) or Fisher’s exact tests was used. For variance comparisons, a least square means analysis for unbalanced samples was applied [35].

#### Modeling approaches

To better understand the influence of the hypothesized relationships at different age levels [36, 37], we separately performed a series of multivariable linear regressions for the physical health and the mental health summary models. The three age groups were entered as dummy variables when performing univariate and multivariate analyses. For each model, covariates were entered as blocks in the following order: demographics (step 1), socioeconomic status (step 2), social factors (step 3), HIV clinical markers (step 4) and unmet basic needs (step 5). To understand the effect of unmet basic needs across subject groups [37], we tested the interaction terms (step 6), which were unmet basic needs x the middle-aged group, and unmet basic needs x the older group. We assessed interaction effects via the visuographic method. We eliminated co-linearity, using the variance inflation factor and eigenvalue [35]. A two-sided *p*-value of less than 0.05 was used for statistically significant variables throughout. All analyses were performed using SAS software 9.3 (Cary, North Carolina, USA).

## Results

### Sample characteristics

Mean age (SD) of the sample was 43.7 (8.4) years, [range 20-70] (Table 1). A majority of respondents (80.4%) in our sample reported being unemployed. Over half (52.0%) had been previously diagnosed with AIDS. The sample mean (SD) for physical health summary scores was 42.6 (10.8) [range14.9-64.6], and the sample mean (SD) for health summary scores was 43.4 (11.9) [range 8.7-68.6]. Role functioning scored lowest, 40.9 (10.4). The highest score was on the pain subscale, 47.8 (9.7).

**Table 1:** Sample characteristics by age group (*N* = 496)

Variables		Age groups			*p* ^a^
	All sample	20-34	35-49	≥ 50	
	*N* = 496	*n* = 70	*n* = 310	*n* = 116	
	%	%	%	%	
Demographic variables					
Male	76.2	52.9	79.4	81.9	<0.001
Caucasian	75.6	65.7	73.6	87.1	0.002
Heterosexual	34.1	54.3	32.9	27.6	<0.001
Economic variables					
No high school degree	20.6	25.7	21.6	14.7	0.15
Unemployed	80.4	74.3	79.7	86.2	0.12
Low income (< 1150 CAD/month)	51.4	61.4	49.0	51.7	0.17
Social variables					
Low support (MOS-SSS < 43)	51.0	51.4	48.7	56.9	0.32
Living alone	50.0	22.9	50.7	64.7	<0.001
Clinical variables					
A history of AIDS diagnosis	52.0	24.3	56.1	57.8	<0.001
CD4 count < 200 cells/mL (*n* = 412)	60.9	28.9	65.5	65.7	<0.001
Years since HIV diagnosis (mean, SD)	11.4 (6.5)	5.3 (4.5)	11.7 (6.0)	14.3 (6.4)	<0.001
Years since starting ART (*n* = 379, mean, SD)	8.1 (5.5)	4.0 (3.6)	7.9 (5.3)	9.7 (5.6)	<0.001
Health-related quality of life					
Physical health summary scores (mean, SD)	42.6 (10.8)	47.5 (9.0)	42.7 (11.0)	39.5 (10.2)	<0.001
Mental health summary scores (mean, SD)	43.4 (11.9)	42.4 (12.5)	43.6 (11.9)	43.5 (11.6)	0.72

^a^*p* of Chi-Square test (two-sided) or one-way ANOVA test. *MOS-SSS*: Medical Outcomes Study Social Support Survey; *ART*: antiretroviral therapy; *SD*: Standard Deviation

Our sample significantly differed among age categories with respect to gender, sexual orientation and ethnicity. Gay or bisexual male participants were older than female same sex or heterosexual participants. There were no significant differences across educational level, employment status, gross income or social support. Compared to the two younger groups, the older group had lower mean physical health summary scores (*p* < 0.001).

### Basic needs

**Fig. 1:**
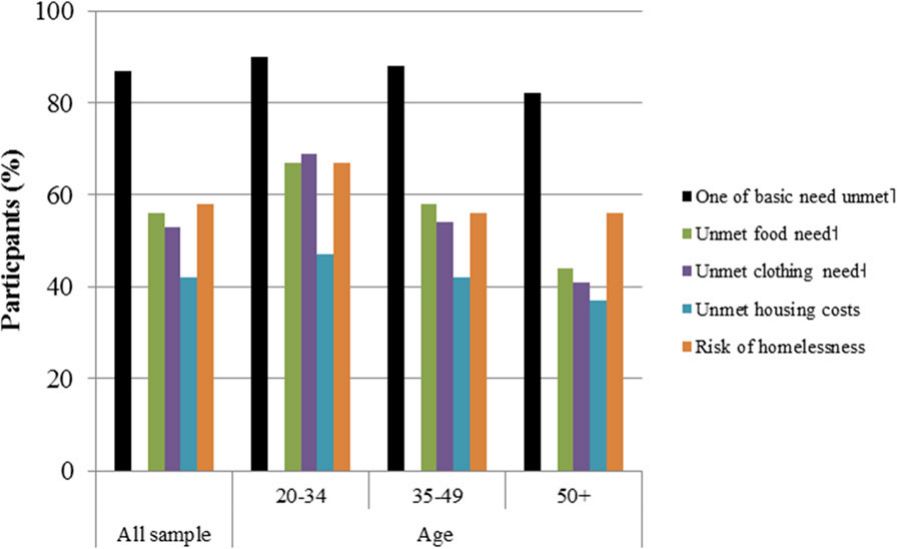
Proportions of each unmet basic need by age group (*N* = 496).Chi-Square tests: ˥ Basic need unmet (p = 0.19); ˦ Unmet food need (p = 0.001); ˧ Unmet clothing need (p = 0.005)

Over 85% of the respondents self-reported at least one unmet basic need. Substantially fewer individuals in the middle-aged and old age groups reported unmet basic needs than did those in the youngest group (Fig. 1) although the results were not statistically significant (*p* = 0.19). When analyzing basic needs across demographics, the prevalence of unmet basic needs did not differ by gender (men, 85.5% vs. women, 90.7%), ethnic group (Caucasians, 88.4% vs. other, 86.1%) or sexual preference (non-heterosexuals, 85.3% vs. other, 89.4%). Participants without a high school degree and those with no job at the time of interview were more likely to report unmet basic needs than their peers (93.1% vs. 85.0%, *p* = 0.03 or 88.7% vs. 78.3%, *p* = 0.007; respectively). Among those with personal gross income below 1150 CAD/month (51.4%), the two older groups were less likely than the youngest group to report unmet needs (*p* = 0.08). Among those who were unemployed, the two older groups were less likely to report unmet needs than the youngest group, but the results did not quite reach statistical significance. Among those who had not finished high school (20.6%), the oldest group (76%) was less likely to report unmet needs than the two younger groups (*p* = 0.01, Fisher’s exact).

#### Univariate analyses of HRQoL

The presence of unmet basic needs was associated with both poorer physical health and poorer mental health summary scores (β = − 6.4, *p* < 0.001 and β = − 8.2, *p* < 0.001, respectively, Table 2). The middle-aged group, the older group, unemployed individuals, Caucasians, those with a previous diagnosis of AIDS and with a longer duration since HIV diagnosis, all had significantly lower physical health summary scores. Being female, unemployed, heterosexual, having low social support and a personal income below 1150 CAD/month were all significantly associated with lower mental health summary scores.

**Table 2:** Univariate regression analyses of physical health and mental health summary scores. (*N* = 496)

Variables	Physical health summary			Mental health summary		
	β	(95% CI)	*p*	β	(95% CI)	*p*
Demographic variables						
Age 20-34	Ref.			Ref.		
Age 35-49	**−4.80**	(−7.54, −2.06)	<0.001	1.28	(−1.81, 4.38)	0.415
Age 50+	**−7.97**	(−11.1, −4.83)	<0.001	1.14	(−2.40, 4.68)	0.528
Female	−1.39	(−3.63, 0.84)	0.222	**−4.08**	(−6.52, −1.64)	0.001
Caucasian	**−2.82**	(−5.02, −0.61)	0.012	−0.78	(−3.23, 1.66)	0.529
Heterosexual	−1.13	(−3.14, 0.88)	0.270	**−3.03**	(−5.23, −0.83)	0.007
Economic variables						
No high school degreedegree	**−2.76**	(−5.11, −0.42)	0.021	**−3.49**	(−6.08, −0.91)	0.008
Unemployed	**−7.44**	(−9.75, −5.13)	<0.001	**−6.23**	(−8.82, −3.64)	<0.001
Low income (< 1150 CAD/month)	−1.15	(−3.06, 0.75)	0.235	**−3.23**	(−5.31, −1.14)	0.003
Social variables						
Low support (MOS-SSS < 43)	**−3.39**	(−5.27, −1.51)	<0.001	**−6.96**	(−8.96, −4.95)	<0.001
Living alone	**−2.10**	(−4.0, −0.20)	0.030	−1.98	(−4.07, 0.11)	0.064
Clinical variables						
A history of AIDS diagnosis	**−5.07**	(−6.92, −3.22)	<0.001	**−3.09**	(−5.18, −1.01)	0.004
CD4 count < 200 cells/mL [*n* = 412]	**−2.48**	(−4.58, −0.38)	0.021	0.56	(−1.82, 2.93)	0.646
Years since HIV diagnosis	**−0.15**	(−0.29, −0.00)	0.045	**0.22**	(0.06, 0.38)	0.007
Years since starting ART [*n* = 379]	**−0.20**	(−0.40, −0.01)	0.041	0.21	(−0.01, −0.43)	0.063
Basic needs variable						
Unmet basic needs	**−6.58** ^a^	(−9.31, −3.86)	<0.001	**−8.24** ^b^	(−11.25, −5.24)	<0.001

Bold values are significant of *p* < 0.05. ^a^Estimates (β) unmet needs: food = −3.95, clothing = −3.81, housing costs = −4.23 (all, *p* < 0.001), risk of homelessness = −1.66 (*p* > 0.05). ^b^Estimates (β) unmet needs: food = −5.9, clothing = −5.4, housing costs = −5.43 or risk of homelessness = −3.64 (all, *p* < 0.001), *CI*: Confidence Interval; *MOS-SSS*: Medical Outcomes Study Social Support Survey; *ART*: antiretroviral therapy

### Multivariate analyses of HRQoL

We employed a series of hierarchical multiple regression analyses to evaluate the effect of unmet basic needs on physical health summary scores (Table 3). We entered the following variables in order: demographics (step 1), socioeconomic status (step 2), social factors (step 3) and HIV clinical markers (step 4). After entering unmet basic needs (step 5), the proportion of variance increased from 19.2% to 22.1%. After entering the two interaction terms (step 6), the amount of variance increased significantly to 23.5%.

**Table 3:** Hierarchical multivariate regression models for physical health summary scores. (*N* = 496)

	Step 1	Step 2	Step 3	Step 4	Step 5	Step 6
	β	β	β	β	β^*^	β
Intercept	51.48^a^	56.83^a^	58.38^a^	58.67^a^	63.10^a^	54.10^a^
Block 1						
Age group						
20-34 (Ref.)	1	1	1	1	1	1
35-49	−5.52^a^	−5.12^a^	−5.21^a^	−4.28^b^	−4.18^b^	5.33
50+	−8.39^a^	−7.63^a^	−7.46^a^	−6.58^a^	−6.80^a^	4.95
Female	−2.32	−2.65^d^	−3.06^c^	−3.08^c^	−2.88^c^	−2.95^c^
Caucasian	−3.14^b^	−3.31^b^	−3.60^b^	−3.43^b^	−3.37^b^	−3.43^b^
Heterosexual	−1.57	−1.0	−0.62	−0.72	−0.79	−0.72
Block 2						
No high school degree		−1.79	−2.06^d^	−1.90^d^	−1.54	−1.62
Unemployed		−6.91^a^	−6.60^a^	−6.04^a^	−5.46^a^	−5.50^a^
Low income (< 1150 CAD/month)		0.30	0.49	0.43	0.39	0.29
Block 3						
Low support (MOS-SSS < 43)			−3.17^a^	−3.16^a^	−2.94^b^	−2.95^b^
Living alone			−0.03	0.02	−0.19	−0.26
Block 4						
A history of AIDS diagnosis				−3.64^a^	−3.64^a^	−3.59^a^
Years since HIV diagnosis				0.02	−0.002	−0.005
Block 5						
Unmet basic needs					−5.53^a*^	4.67
Block 6						
Unmet basic needs X age 35-49						−10.56^c^
Unmet basic needs X age 50+						−13.22^b^
*R* ^2^	0.073	0.144	0.165	0.192	0.221	0.235
*F* Change	7.725^a^	13.537^a^	6.110^b^	7.880^a^	18.049^a^	4.423^b^
*F* Test	7.73^a^	10.28^a^	9.61^a^	9.55^a^	10.52^a^	9.83^a^

^a^*p* < 0.001, ^b^*p* < 0.01, ^c^*p* < 0.05, ^d^*p* approached the significance < 0.05. CD4 count < 200 cells/mL was not included because it was insignificantly associated with physical health summary scores (*p* = 0.566). Years since starting ART was excluded because of co-linearity with the variable, years since HIV diagnosis. *Further multivariable linear regression models were performed for each basic need, estimated coefficient (β) unmet needs: food = −3.65, clothing = −3.72, housing cost = −3.67 (all, *p* < 0.001) or risk of homelessness = −1.04 (*p* > 0.05)

To evaluate the effect of unmet basic needs on mental health summary scores, we employed a series of hierarchical multiple regression analyses as shown in Table 4. We repeated step 1 to 4 as we did previously. In step 5, after adding the unmet basic needs, the variance increased significantly from 19.2% to 22.4%. In step 6, after adding both interaction terms as we did before, the amount of variance increased significantly to 24.1%.

**Table 4:** Hierarchical multivariate regression models for mental health summary scores. (*N* = 496)

	Step 1	Step 2	Step 3	Step 4	Step 5	Step 6
	β	β	β	β	β	β
Intercept	46.40^a^	51.99^a^	55.39^a^	54.32^a^	59.46^a^	47.94^a^
Block 1						
Age group						
20-34 (Ref.)	0	0	0	0	0	0
35-49	0.18	0.26	0.14	−0.39	−0.27	12.66^b^
50+	0.18	0.61	1.09	−0.04	−0.24	13.23^b^
Female	−3.49^c^	−3.73^c^	−4.67^b^	−4.61^b^	−4.38^b^	−4.47^b^
Caucasian	−2.29^d^	−2.51^d^	−3.12^c^	−3.20^b^	−3.13^b^	−3.23^b^
Heterosexual	−1.63	−1.13	−0.41	0.06	−0.03	0.06
Block 2						
No high school degree		−1.59	−2.17^d^	−2.30^d^	−1.87	−1.89
Unemployed		−5.56^a^	−4.87^a^	−4.55^a^	−3.87^b^	−3.91^b^
Low income (< 1150 CAD/month)		−1.74	−1.26	−1.14	−1.19	−1.30
Block 3						
Low social support (MOS-SSS < 43)			−6.85^a^	−6.91^a^	−6.65^a^	−6.66^a^
Living alone			−0.37	−0.20	−0.48	−0.53
Block 4						
A history of AIDS diagnosis				−2.98^b^	−2.98^b^	−2.90^b^
Years since HIV diagnosis				0.24^b^	0.22^c^	0.21^c^
Block 5						
Unmet basic needs					−6.40^a*^	6.60
Block 6						
Unmet basic needs X age 35-49						−14.39^b^
Unmet basic needs X age 50+						−15.09^b^
*R* ^2^	0.029	0.083	0.165	0.192	0.224	0.241
*F* Change	2.889^b^	9.578^a^	23.922^a^	8.050^a^	19.978^a^	5.410^b^
*F* Test	2.89^b^	5.49^a^	9.59^a^	9.57^a^	10.72^a^	10.18^a^

^a^*p* < 0.001, ^b^*p* < 0.01, ^c^*p* < 0.05, ^d^*p* approached the significance < 0.05. CD4 count < 200 cells/mL was not included because it was insignificantly associated with mental health summary scores (*p* = 0.407). Years since starting ART was excluded because of co-linearity with the variable, years since HIV diagnosis. *Further multivariable linear regression models were performed for each basic need, estimated coefficient (β) unmet needs: food = −4.10, clothing = −3.39, housing cost = −4.34 or risk of homelessness = −3.07 (all, *p* < 0.001)

#### Interaction terms

**Fig. 2:**
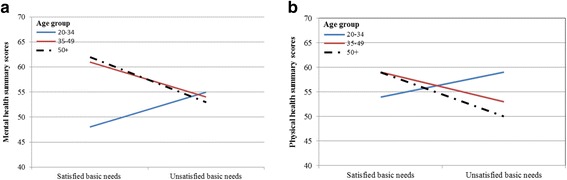
Adjusted models of interaction terms between unmet basic needs and age groups (*N* = 496). **a** Multivariate regression model: The effect of unmet basic needs on physical health summary score was steeper in the oldest group than the middle-aged group (*F* test = 4.72, *p* = 0.009, R^2^ = 0.02). **b** Multivariate regression model: Mental health summary score was reduced by the effect of unmet basic needs, with the slope a bit steeper in the oldest group than in the middle-aged group (*F* test = 5.60, *p* = 0.004, R^2^ = 0.02)

We noted that unmet needs lowered physical health scores to a greater degree in the oldest group than they did in the middle-aged group (Fig. 2a). On mental health summary scores, the slope was a bit steeper in the oldest group compared to the middle-aged group as a result of the effect of unmet needs (Fig. 2b).

## Discussion

Our primary hypothesis was that unmet basic needs would be significantly and negatively associated with lower physical health or mental health quality of life summary scores even after controlling for potential confounders. Also, we predicted that the impact of unmet basic needs would vary across age groups, and increase with age. We found a very high prevalence of unmet basic needs in our HIV study participants in Ontario, Canada. We noted that age moderated the influence of unmet basic needs on HRQoL.

Our conceptualization of unmet basic needs as a predictor of poor health and well-being is consistent with previous studies related to basic subsistence needs in HIV-infected adults [1–3]. The very high prevalence of unmet basic needs among people living with HIV in our study may have been magnified by the nature of our sample, which was purposefully recruited to represent a population with unstable housing. This notwithstanding, it is important to note that, when examining the influence of demographics or socioeconomic status, the proportion of unmet basic needs did not differ by gender, race or sexual orientation. This suggests that problems meeting basic needs is an across-the-board challenge for people living with HIV in our province.

The fact that there was a trend (not quite reaching statistical significance) for the prevalence of unmet basic needs in the two older groups to be lower than that in the younger participants, can perhaps be explained by the demographic and socioeconomic profiles of the two older groups, which were very similar to study populations described in other reports [15, 38, 39]. Our two older groups were well-educated and enjoyed relatively high personal incomes. The high rate of unemployment in this older population was probably the result of their chronic illness. However, participants belonging to the two older groups who reported lower socioeconomic status had a significantly lower prevalence of unmet basic needs than the youngest group. As Joyce et al. [39] noted, older white and gay men with HIV enjoy greater financial stability than their younger peers. Previous studies have shown that older individuals with HIV have better adherence [40] and achieve a more rapid virologic response to antiretroviral treatments than younger peers [41]. Older adults with HIV, compared to younger adults, also tend to show greater resilience to stress [42, 43]. This may increase physical, emotional and functional well-being, a sign of successful aging [44, 45].

We found that age moderated the main effect of unmet basic needs on both physical health and mental health summaries. The effect of unmet basic needs on mean physical health summary scores was much greater in the oldest group than in the middle-aged group. In contrast, the effect of unmet basic needs on mean mental health summary scores was essentially similar. These findings have health policy implications and offer insights into public health priorities targeting HIV-positive aging populations.

Our findings that the presence of unmet basic needs reduced mean physical health and mental health summary scores in the middle-aged and the oldest groups also have clinical importance. Studies have shown that the effect of age has a negative impact on physical health among HIV-positive populations [24, 46]. In our physical health summary model, when a basic need was not met, a decrement of 6- and 9-points in the middle-aged and the oldest groups, respectively, was observed. This is because the oldest group had significantly lower scores than the middle-aged group, and they were even lower than those of the youngest group, in all physical health subscales.

Studies have reported that age is associated with relatively good mental health quality of life [42, 47]. In our mental health summary model, when a basic need was not met, a decrement of 7- and 9- points was observed in the middle-aged and oldest groups, respectively. This suggests that the effect of unmet basic needs influences the mean mental health scores of the two older groups. The insignificant difference between satisfied and unsatisfied basic needs for mean scores of HRQoL in the younger group does not necessarily imply that unmet basic needs will not influence this group. Rather, as these participants age, their health may be expected to deteriorate should they continue to face unmet basic needs. Also, this group was relatively smaller compared to the middle-aged and the oldest groups.

There are some limitations to note in our study. A validated instrument for measuring basic needs would have improved our findings. It is also evident that, using cross-sectional data, we cannot infer a direct causal pathway between unmet basic needs and poor HRQoL but can only point out the association. In addition, because the sample participants were mostly individuals seeking services at community-based AIDS service organizations, our results may not generalize to all HIV-positive persons in Ontario or beyond. Our results, of course, rely on the accuracy of self-reporting on the part of the participants. We also did not control for depression. The scores for this variable would have correlated with all dimensional subscales, particularly with the mental health summary components. Thus, they would have biased the results in favour of our hypothesis.

## Conclusions

The strength of our study is that it confirms the relationship between unmet basic needs and physical and mental health quality of life by age group, using reliable statistical tests. We recommend that future studies: 1) develop a valid instrument for measuring basic needs; 2) determine the causes of the high prevalence of unmet basic needs in this population beyond demographic and socioeconomic factors; 3) examine the longitudinal influence of unmet basic needs on HRQoL, using a variety of instruments, not only the MOS-HIV; and 4) determine the influence of unmet basic needs on comorbidities and life expectancy.

## Footnotes

1 *RH* is being at risk of homelessness, *inc* is a personal gross income per month (Question 50), and *mor* is an amount rent or mortgage payment per month (Question 57).
